# Association between the C-reactive protein/albumin ratio and mortality in older Japanese patients with dysphagia

**DOI:** 10.3389/fnut.2024.1370763

**Published:** 2024-06-27

**Authors:** Chunhong Guo, Pingping Zheng, Shiyang Chen, Lin Wei, Xiuzhen Fu, Youyuan Fu, Tianhong Hu, Shaohua Chen

**Affiliations:** ^1^The Second Clinical College of Guangzhou University of Chinese Medicine, Guangzhou, China; ^2^Division of Spine Center, The Second Affiliated Hospital of Guangzhou University of Chinese Medicine (Guangdong Provincial Hospital of Chinese Medicine), Guangzhou, China; ^3^Nursing Department, The Second Affiliated Hospital of Guangzhou University of Chinese Medicine, Guangzhou, China; ^4^Department of Breast Oncology, The Second Affiliated Hospital of Guangzhou University of Chinese Medicine, Guangzhou, China

**Keywords:** dysphagia, mortality, C-reactive protein, albumin, C-reactive protein-to-albumin ratio

## Abstract

**Background:**

C-reactive protein-to-albumin ratio (CRP/ALB) has been proven to represent a biomarker for predicting prognosis in many groups of patients with severe diseases. However, few studies have investigated the association between CRP/ALB and mortality in Japan older people with dysphagia patients.

**Objective:**

This retrospective cohort study aimed to assess the prognostic value of C-reactive protein/albumin ratio (CAR) in older Japanese patients with dysphagia.

**Methods:**

We analyzed data from 253 patients diagnosed with dysphagia at a single center between January 2014 and January 2017. Cox regression analysis was used to compare the mortality rates across the CAR tertiles. Subgroup analyses were conducted, and Kaplan–Meier curves were used to determine the median survival times.

**Results:**

The study included 154 female and 99 male patients, with a median age of 83 years. After adjusting for all covariates, the multivariable Cox regression analysis revealed a significant association between increasing CAR (HR = 1.19, 95% CI: 1.03–1.37, *P* = 0.022) and the risk of mortality. Compared to the reference group T1 (< 0.149), the adjusted hazard ratios for T2 (0.149–0.815) and T3 (> 0.815) were 1.75 (95% CI: 1.07–2.87, *P* = 0.027) and 2.15 (95% CI: 1.34–3.46, *P* = 0.002), respectively. Kaplan–Meier curves indicated median survival times of 864, 371, and 223 days for T1, T2, and T3, respectively.

**Conclusion:**

The C-reactive protein/albumin ratio was positively related to mortality in Japan older people with dysphagia patients. There was no interaction for the subgroup analysis. The result was stable.

## 1 Introduction

Dysphagia, a prevalent condition among older adults, has been found the estimated prevalence in 8% of the global population (1, 2), with roughly 1 million new cases identified each year in the United States, which is equal to 1 out of every 25 adults (3). For older individuals living in communities, the occurrence of dysphagia is approximately 15%, while in patients admitted to hospitals, it is closer to 30% (4, 5). Dysphagia is most commonly seen in elderly patients with neurological conditions and dementia, with rates of prevalence at 64 and 80%, respectively (6). Moreover, oropharyngeal dysphagia can also affect 70 to 80% of patients after radiotherapy for nasopharyngeal cancer (7) and 1 to 79% of patients with anterior cervical fusion (8). Individuals diagnosed with dysphagia face a range of health hazards, including malnutrition, pneumonia, dehydration, elevated mortality rates, as well as a higher probability of requiring long-term care (9–11) and increased medical expenses (12). Notably, a hospital-based study revealed that patients with dysphagia were 1.7 times more prone to mortality than their counterparts without this condition (12). Therefore, prioritizing the evaluation and treatment of dysphagia is imperative.

C-reactive protein (CRP) and albumin (ALB) are hepatically synthesized biochemical markers present in the bloodstream that serve as indicators of inflammation and nutritional status, respectively. The concept of the C-reactive protein-to-albumin ratio (CAR) was first introduced by Fairclough et al. (13) and has since been recognized as a prognostic factor for disease outcomes, particularly in the older adult population (13). Elevated CAR values have consistently been associated with increased mortality rates and unfavorable prognoses across various patient cohorts, including those afflicted with cancer, individuals in intensive care units (ICUs), patients with pancreatitis, and those with COVID-19 (14–24).

However, studies have indicated a persistent absence of comprehensive guidelines for the management of dysphagia in the older adult population. Consequently, early assessment plays a pivotal role in prognosticating unfavorable outcomes associated with dysphagia. While prior research has established the predictive value of CRP/ALB in various diseases, the current body of literature provides limited evidence regarding the correlation between CAR and mortality in individuals with dysphagia. Factors predicting mortality in patients with dysphagia are still under investigation, and a comprehensive consensus on this matter is lacking. Consequently, this study aimed to examine whether the CAR was independently associated with mortality in older individuals with dysphagia residing in Japan.

## 2 Materials and methods

### 2.1 Data source

The data for this study were sourced from the Dryad Digital Repository,^1^ a platform that provides users with unrestricted access to and retrieval of the original data. In compliance with the Dryad Terms of Service, we referred to the Dryad data package titled “Baseline C-reactive protein, albumin level, and life prognosis in Dryad,” available at https://doi.org/10.5061/dryad.gg407h1 for our current investigation. The results of these studies were published in 2019 (25).

### 2.2 Study design and participants

This retrospective cohort study was conducted at a single focusing on older patients with dysphagia who underwent percutaneous endoscopic gastrostomy (PEG) or total parenteral nutrition (TPN) between January 2014 and January 2017. All of the patients in this data, dysphagia was evaluated clinically by a doctor, nurse, and speech-language pathologist, along with assessments through video fluoroscopy. As a result, it was evident that each patient had severe dysphagia. Patients with terminal cancer or those requiring PEG for gastric decompression as well as individuals who underwent PEG before January 2014 were excluded from the study. Data anonymization eliminated the need for informed consent, and all methodologies adhered to the relevant guidelines and regulations. This retrospective study was approved by the Ethical Review Board of Miyanomori Memorial Hospital, which waived the requirement for informed consent.

### 2.3 Procedures

The decision to opt for either PEG feeding or TPN was made through comprehensive discussions involving the patients (or their family members) and clinicians. Appropriate nutrition was administered based on the clinical evaluations conducted by healthcare professionals. Clinical information, including age (26), sex, underlying diseases such as cerebrovascular diseases (26), severe dementia (6), aspiration pneumonia (27), ischemic heart disease (IHD), the presence of non-tunneled central venous catheters (NT.CVC), percutaneous endoscopic gastrostomy (PEG) (25, 28), oral intake recovery, and blood test results were obtained from patients’ medical records. Blood tests were performed within 7 days prior to the commencement of PEG feeding or TPN.

The primary objective of this study was to assess mortality rates following the initiation of the procedure during the designated follow-up period. The formula used in this study was as follows: CAR = CRP/ALB. Patients were categorized into three groups, namely T1 (< 0.149), T2 (0.149–0.815), and T3 (≥ 0.815), based on their CAR at the time of enrollment.

### 2.4 Statistical analysis

Secondary analyses were performed using publicly accessible datasets. Categorical variables are expressed as percentages (%), while continuous variables are presented as mean (SD) or median (IQR). To assess baseline characteristics, a one-way ANOVA was applied to continuous variables, and a chi-square test was conducted to evaluate the statistical differences among the tertiles of the CAR for classified variables. The correlation between CAR and mortality in patients with dysphagia was examined using Cox proportional hazards model. Survival curves were generated using the Kaplan–Meier and log-rank analyses. The likelihood ratio test was used to explore the interactions among the subgroups. All statistical analyses were performed using R^2^ and Free Statistics software version 1.9. A two-sided significance level of *P* < 0.05 (two-sided) was considered, and all reported *p*-values were less than 0.05.

## 3 Results

### 3.1 Study participants and baseline characteristics

Table 1 presents the essential characteristics of the cohort of 253 (99 male and 154 female) patients. The average age of the patients was 83.1 years, with a standard deviation of 9.3. Among them, 180 underwent PEG feeding and 73 underwent TPN. For patients with censored data, the median follow-up duration was 601 days (range, 404–823 days). Notably, significant differences were observed among the three groups in terms of cerebrovascular disease, severe dementia, aspiration pneumonia, oral intake recovery, PEG, and NT-CVC (*P* < 0.05).

**TABLE 1:** Baseline characteristics of patients.

Variables	Total	CAR, T1 (< 0.149)	CAR, T2 (0.149∼0.815)	CAR, T3 (≥ 0.815)	*P*
	(*n* = 253)	(*n* = 84)	(*n* = 84)	(*n* = 85)	
Age (year)	83.1 ± 9.3	81.4 ± 10.8	83.0 ± 9.5	84.8 ± 7.2	0.068
Sex, *n* (%)					0.487
Male	99 (39.1)	29 (34.5)	33 (39.3)	37 (43.5)	
Female	154 (60.9)	55 (65.5)	51 (60.7)	48 (56.5)	
CI, *n* (%)	133 (52.6)	52 (61.9)	45 (53.6)	36 (42.4)	0.038
Dement, *n* (%)	102 (40.3)	23 (27.4)	36 (42.9)	43 (50.6)	0.007
Asp, *n* (%)	94 (37.2)	22 (26.2)	31 (36.9)	41 (48.2)	0.012
IHD, *n* (%)	47 (18.6)	13 (15.5)	12 (14.3)	22 (25.9)	0.103
Hemoglobin	11.0 ± 2.0	11.9 ± 1.8	10.9 ± 2.0	10.1 ± 1.9	< 0.001
Oral, *n* (%)	15 (5.9)	9 (10.7)	3 (3.6)	3 (3.5)	0.097
PEG, *n* (%)	180 (71.1)	74 (88.1)	55 (65.5)	51 (60)	< 0.001
NT.CVC, *n* (%)	26 (10.3)	3 (3.6)	9 (10.7)	14 (16.5)	0.022
Status, *n* (%)					< 0.001
Death	115 (45.5)	56 (66.7)	33 (39.3)	26 (30.6)	
Alive	138 (54.5)	28 (33.3)	51 (60.7)	59 (69.4)	
CAR	0.3 (0.1, 1.1)	0.1 (0.0, 0.1)	0.3 (0.2, 0.5)	1.8 (1.1, 2.8)	< 0.001

CI, cerebrovascular diseases; dement, severe dementia; asp, aspiration pneumonia; IHD, ischemic heart disease; NT.CVC, non-tunneled central venous catheters; PEG, percutaneous endoscopic gastrostomy; oral, oral intake recovery; CAR, C-reactive protein/albumin ratio.

### 3.2 Kaplan–Meier curve

**FIGURE 1:**
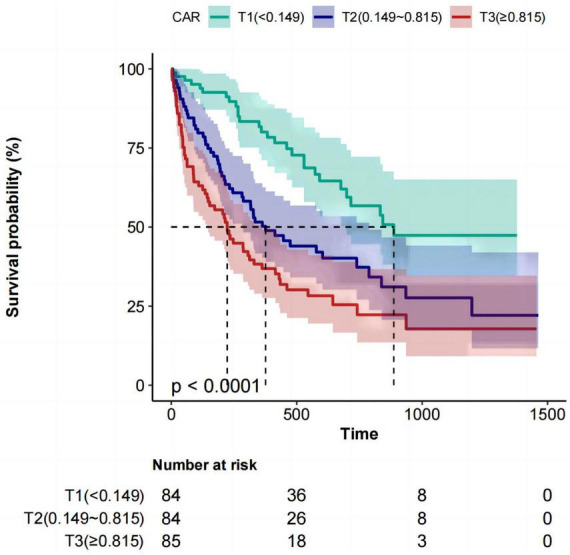
Kaplan–Meier survival analysis for mortality with CAR in three groups. CAR, C-reactive protein-to-albumin ratio.

The Kaplan–Meier curve in Figure 1 indicates that both the T3 and T2 groups demonstrated significantly shorter median survival durations than the T1 group. The respective values for median survival times were 223, 371, and 864 days (*P* < 0.0001).

### 3.3 Association between CAR and mortality in various models

Table 2 illustrates the hazard ratio (HR) and 95% confidence intervals (95% Cl) associated with the risk of mortality in patients with dysphagia based on CAR. There are four models for CAR as continuous and categorical variables. No variables to be chosen to adjust in modelI. Variables of demographics (age, sex) and past medical history (cerebrovascular diseases, severe dementia, aspiration pneumonia, IHD) were chosen to adjust in modelII. All the variables that we included (age, sex, IHD, cerebrovascular diseases, severe dementia, aspiration pneumonia, PEG, Hb, NT.CVC, oral intake recovery) to be adjusted in model III. The results of these models were shown in Table 2. The risk of mortality exhibited an upward trend as CAR increased in the univariable Cox regression analysis (HR = 1.28, 95% CI: 1.14–1.44, *P* < 0.001). Upon adjusting for all covariates in the multivariable Cox regression analysis, the HR was 1.19 (95% CI: 1.03–1.37, *P* = 0.022). When compared to the lowest CAR group (T1 < 0.149), the adjusted HR values for CAR and mortality in the T2 (0.149–0.815) and T3 (≥ 0.815) groups were 1.75 (95% CI: 1.07–2.87, *P* = 0.027) and 2.15 (95% CI: 1.34–3.46, *P* for trend = 0.002), respectively.

**TABLE 2:** Association between CAR and mortality in different models.

Variable	Model I		Model II		Model III	
	HR (95% CI)	*P*	HR (95% CI)	*P*	HR (95% CI)	*P*
CAR	1.28 (1.14∼1.44)	< 0.001	1.19 (1.05∼1.36)	0.006	1.19 (1.03∼1.37)	0.022
CAR (T1, < 0.149)	1 (Ref)		1 (Ref)		1 (Ref)	
CAR (T2, 0.149∼0.815)	2.2 (1.39∼3.49)	0.001	2.34 (1.45∼3.76)	< 0.001	1.75 (1.07∼2.87)	0.027
CAR (T3, ≥ 0.815)	3.35 (2.13∼5.27)	< 0.001	2.97 (1.87∼4.73)	< 0.001	2.15 (1.34∼3.46)	0.002
*P* for trend		< 0.001		< 0.001		0.002

Model I: non-adjusted.

Model II: adjusted for age, sex, cerebrovascular diseases, severe dementia, aspiration pneumonia, ischemic heart disease.

Model III: adjusted for age, sex, cerebrovascular diseases, severe dementia, aspiration pneumonia, ischemic heart disease, non-tunneled central venous catheters, percutaneous endoscopic gastrostomy, oral intake recovery, hemoglobin. CAR, C-reactive protein-to-albumin ratio.

### 3.4 Subgroup analyses

**FIGURE 2:**
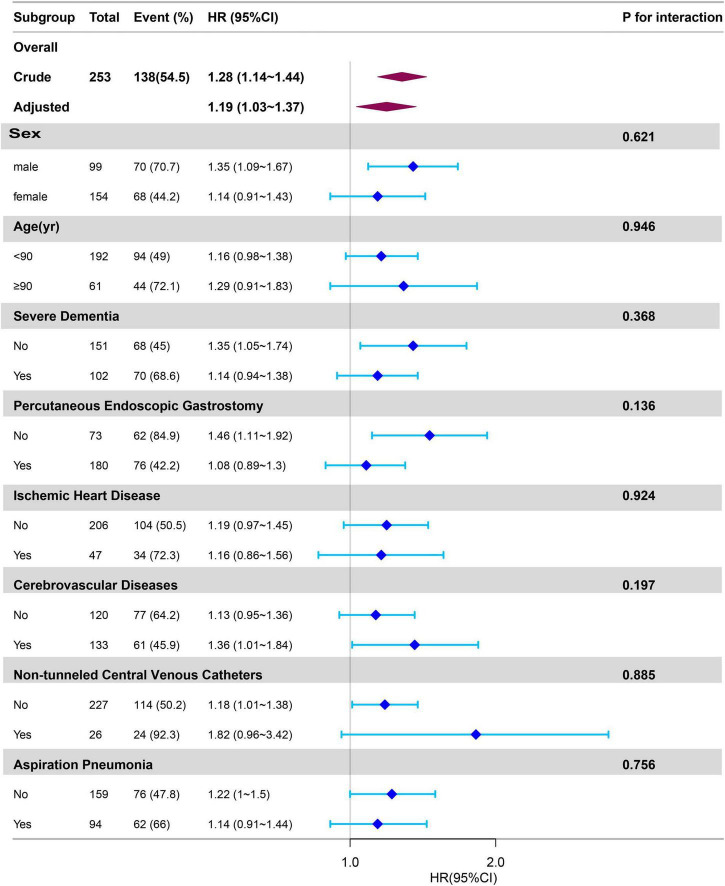
Subgroup analyses of CAR associated with mortality. Hazard ratios (HRs) were adjusted for age, sex, cerebrovascular diseases, severe dementia, aspiration pneumonia, ischemic heart disease, non-tunneled central venous catheters, percutaneous endoscopic gastrostomy, oral intake recovery, hemoglobin. CAR, C-reactive protein-to-albumin ratio.

Subgroup and interaction analyses were performed to assess the consistency of the correlation between CAR and dysphagia-related mortality (Figure 2). After have been adjusted for all the covariates (age, sex, IHD, cerebrovascular diseases, severe dementia, aspiration pneumonia, PEG, Hb, NT.CVC, oral intake recovery) that we included, and during subgroup analysis, if the subgroup analysis variable is a categorical variable, it is excluded from the analysis. There was no interaction for eight subgroups. The result was stable and depicted in Figure 2.

## 4 Discussion

Our study revealed a significant positive correlation between CAR and mortality. Specifically, when treating CAR as a continuous variable, our unadjusted model shows a noteworthy correlation between CARs and the risk of mortality in patients with dysphagia (HR: 1.28, 95% CI: 1.14–1.44, *P* < 0.001). Furthermore, even after accounting for all covariates, the adjusted model indicated a significant association (HR = 1.19, 95% CI: 1.03–1.37, *P* = 0.022). When CAR was considered a categorical variable, a significant association with dysphagia-related mortality was observed in the crude model, with the T3 group showing a two-fold increase in risk compared with the T1 group. After adjusting for all covariates in the multivariable analyses, the statistical significance of the association between CAR and dysphagia-related mortality persisted. Specifically, in the T2 and T3 groups, the risk of all-cause mortality was 1.75 and 2.15 times higher, respectively, than that in the T1 group.

The primary focus of this study was the CAR, which functions as an indicator of both inflammation and nutritional status in patients with dysphagia (29). The elevated mortality rate observed in older patients with dysphagia is closely associated with malnutrition (30). Malnutrition detrimentally affects the immune system functionality by impeding the proper functioning of immune cells and the body’s ability to effectively combat infections and regulate inflammatory responses. In addition, malnutrition amplifies oxidative stress and the generation of inflammatory mediators, thereby instigating inflammatory reactions and exacerbating the severity of various ailments. It has the potential to induce tissue impairment and dysbiosis of the gastrointestinal microbiota, thereby impacting the health of patients. Consequently, in the context of preventing and managing geriatric dysphagia, it becomes imperative to enhance the nutritional status through the provision of sufficient nutrients to bolster immune functionality and tissue integrity, mitigate the incidence of inflammation, and ultimately curtail mortality rates. The administration of nutritional supplements via enteral or intravenous routes presents a substantial potential risk factor for infection.

In recent years, CAR has garnered recognition as a novel prognostic factor for various diseases such as non-small cell lung cancer (14), hepatocellular carcinoma (18), pancreatic cancer (24), esophageal squamous cell cancer (16), critically ill patients (15), hypo-pharyngeal and laryngeal cancer (17), renal cell carcinoma (19), acute mesenteric ischemia (20), hip fractures In older individuals (21), COVID-19 (22), intrahepatic cholangiocarcinoma (23), myocardial infarction (31) and gastric cancer (32). These studies compared single indicators such as CRP or ALB, and the composite indicator CAR was more advantageous in predicting patient prognosis. A study about the prognosis of 387 patients with non-small cell lung cancer revealed that after categorizing patients based on CAR tertiles, those in the highest CAR group had a 4.14 times higher risk of death compared to those in the lowest CAR group (14). And our study revealed that the group with the highest CAR exhibited a mortality rate 2.15 times greater than that of the lowest CAR. Two studied showed the same trend. There was the difference about the HR as the disease and sample size. This finding provides additional evidence supporting the positive correlation between CAR and dysphagia-related mortality. However, it is crucial to acknowledge the divergent research in this field.

Shafigh et al. (33) proposed the need for additional investigations to establish the predictive capacity of CAR for disease prognosis or mortality. A thorough examination of conflicting studies led us to posit that the divergent outcomes may be attributed to various factors. Primarily, studies opposing our findings focused on ICU patients from a prominent hospital in Tehran, suggesting potential differences in patient characteristics and demographics. Notably, the studies under consideration had relatively small sample size, encompassing only 55 cases. This limitation could potentially restrict the applicability of their findings to a broader population.

The recent guideline emphasizes the importance of promptly evaluating and addressing dysphagia to prevent negative impacts on an individual’s quality of life. These impacts include dehydration, malnutrition, and aspiration pneumonia (34). Several studies (27, 35–39) have also revealed that aspiration pneumonia is a common complication in elderly patients with dysphagia, and it serves as an independent risk factor for death. Furthermore, researches (35, 40) indicated that more than half of dysphagia patients experience complications such as malnutrition and dehydration due to eating disorders, which worsen the progression of the disease. And a study have identified that albumin (ALB) < 3.5 g/L was an independent risk factor for predicting poor prognosis in patients with dysphagia (38).

Our study found that the CRP to ALB ratio is positively correlated with mortality in dysphagia patients, which can serve as a practical tool for evaluating patient outcomes in clinical practice. CRP and ALB are markers used to assess inflammation and nutritional status. High levels of CRP indicate the need for monitoring potential aspiration pneumonia, while low levels of ALB suggest a risk of malnutrition in dysphagia patients. Regular monitoring of changes in CRP/ALB levels allows for prompt detection of fluctuations in patients’ conditions during dysphagia management. As the global population ages, there is an increasing number of elderly dysphagia patients, often resulting from stroke (27, 36) or orthopedic surgeries such as hip fractures (38, 41) or anterior cervical fusion (42, 43). It is important to note that CRP and ALB are standard blood indicators commonly used in clinical practice and do not incur additional costs for patients. Our findings provide healthcare professionals with a foundation for assessing the prognosis of elderly dysphagia patients and support the wider implementation of these measures in clinical settings to improve patient care and outcomes.

Nevertheless, it is important to acknowledge the limitations of the present study. First, the study was conducted at a single center in Japan and was retrospective, potentially compromising its accuracy compared to more robust multicenter prospective studies from different countries. Second, we must recognize the potential presence of selection bias in our study due to the reliance on a solitary measurement of CRP and serum ALB within a 7-day timeframe upon hospitalization without subsequent follow-up measurements. Consequently, the potential effect of CRP and serum ALB levels at varying time intervals on the desired outcomes was precluded. Third, this is the secondary analysis of pre-existing data from an online repository. We didn’t design or control the data collection methodology.

## Conclusion

The C-reactive protein/albumin ratio was positively related to mortality in Japan older people with dysphagia patients. There was no interaction for the subgroup analysis. The result was stable.

## Data availability statement

The original contributions presented in this study are included in this article/supplementary material, further inquiries can be directed to the corresponding author.

## Ethics statement

The studies involving humans were approved by the Ethical Review Board of Miyanomori Memorial Hospital. The studies were conducted in accordance with the local legislation and institutional requirements. The participants provided their written informed consent to participate in this study. Written informed consent was obtained from the individual(s) for the publication of any potentially identifiable images or data included in this article.

## Author contributions

CG: Conceptualization, Data curation, Formal analysis, Writing – original draft, Writing – review & editing. PZ: Data curation, Formal analysis, Writing – review & editing. ShiC: Formal analysis, Writing – review & editing. LW: Methodology, Writing – review & editing. XF: Writing – review & editing. YF: Resources, Writing – review & editing. TH: Funding acquisition, Writing – review & editing. ShaC: Project administration, Supervision, Writing – review & editing.

## Footnotes

1
https://datadryad.org/

2
http://www.R-project.org
